# Virtual reality for pre-procedural planning of valve-in-valve transcatheter aortic valve implantation

**DOI:** 10.1093/ehjdh/ztaf024

**Published:** 2025-03-25

**Authors:** Dominika Kanschik, Jafer Haschemi, Kathrin Klein, Oliver Maier, Stephan Binneboessel, Ursala Tokhi, Shazia Afzal, Patrick W Serruys, Tsung-Ying Tsai, Gerald Antoch, Artur Lichtenberg, Christina Ballázs, Dmytro Stadnik, Maximilian Scherner, Malte Kelm, Tobias Zeus, Christian Jung

**Affiliations:** Department of Cardiology, Pulmonology, and Vascular Medicine, Medical Faculty, University Hospital and Heinrich-Heine University, Germany; Department of Cardiology, Pulmonology, and Vascular Medicine, Medical Faculty, University Hospital and Heinrich-Heine University, Germany; Department of Cardiology, Pulmonology, and Vascular Medicine, Medical Faculty, University Hospital and Heinrich-Heine University, Germany; Department of Cardiology, Pulmonology, and Vascular Medicine, Medical Faculty, University Hospital and Heinrich-Heine University, Germany; Department of Cardiology, Pulmonology, and Vascular Medicine, Medical Faculty, University Hospital and Heinrich-Heine University, Germany; Department of Cardiology, Pulmonology, and Vascular Medicine, Medical Faculty, University Hospital and Heinrich-Heine University, Germany; Heartcenter Trier, Krankenhaus der Barmherzigen Brueder, Trier, Germany; CORRIB Research Centre for Advanced Imaging and Core Lab, University of Galway, Galway, Ireland; CORRIB Research Centre for Advanced Imaging and Core Lab, University of Galway, Galway, Ireland; Department of Diagnostic and Interventional Radiology, Medical Faculty, University Hospital and Heinrich-Heine University, Duesseldorf, Germany; Department of Cardiac Surgery, Medical Faculty, University Hospital and Heinrich-Heine University, Duesseldorf, Germany; Cardiovascular Research Institute Duesseldorf (CARID), Medical Faculty, Heinrich-Heine University, Duesseldorf, Germany; Department of Cardiac Surgery, Medical Faculty, University Hospital and Heinrich-Heine University, Duesseldorf, Germany; Department of Cardiac Surgery, Medical Faculty, University Hospital and Heinrich-Heine University, Duesseldorf, Germany; Department of Cardiac Surgery, Medical Faculty, University Hospital and Heinrich-Heine University, Duesseldorf, Germany; Department of Cardiology, Pulmonology, and Vascular Medicine, Medical Faculty, University Hospital and Heinrich-Heine University, Germany; Cardiovascular Research Institute Duesseldorf (CARID), Medical Faculty, Heinrich-Heine University, Duesseldorf, Germany; Department of Cardiology, Pulmonology, and Vascular Medicine, Medical Faculty, University Hospital and Heinrich-Heine University, Germany; Department of Cardiology, Pulmonology, and Vascular Medicine, Medical Faculty, University Hospital and Heinrich-Heine University, Germany; Cardiovascular Research Institute Duesseldorf (CARID), Medical Faculty, Heinrich-Heine University, Duesseldorf, Germany

**Keywords:** Valve-in-valve transcatheter aortic valve implantation, Virtual reality, Cardiac computed tomography, Imaging

## Abstract

**Aims:**

Valve-in-valve transcatheter aortic valve implantation (ViV-TAVI) has proven to be an effective treatment option for high-risk patients with degenerated surgical bioprosthetic aortic valves. Multislice computed tomography (MSCT) analysis, the current gold standard for procedural planning, has certain limitations. Virtual reality (VR) could optimize pre-procedural planning by delivering three-dimensional (3D) patient-specific information. This study aimed to investigate the feasibility of visualizing the bioprosthesis and adjacent structures with VR, as well as the accuracy and reproducibility of VR measurements and their advantages and limitations in planning ViV-TAVI.

**Methods and results:**

The visualizations and measurements were performed using 3mensio software and VR software by analysts blinded to the results of the other software based on MSCT data from 20 patients who underwent ViV-TAVI interventions. Moreover, eight physicians graded numerous aspects of pre-procedural ViV-TAVI planning with and without VR visualizations. The analysis showed no significant differences between the measurements and strong correlations with correlation coefficients between 0.874 and 0.994, *P* < 0.001. Moreover, good-to-excellent intra- and interobserver reliability with intraclass correlation coefficient values between 0.897 and 0.986 was documented. The qualitative analysis showed that 3D visualization using VR facilitates assessing the spatial relationships between the structures. Furthermore, VR enabled a superior visual understanding of the bioprosthesis and the distances between the virtual prosthesis and the coronaries as well as the sinotubular junction.

**Conclusion:**

Virtual reality can be a valuable addition to the pre-procedural planning of ViV-TAVI interventions, thanks to detailed 3D visualization and precise measurements. Further studies are needed to assess the impact on patient outcomes.

## Graphical Abstract

**Graphical Abstract:**
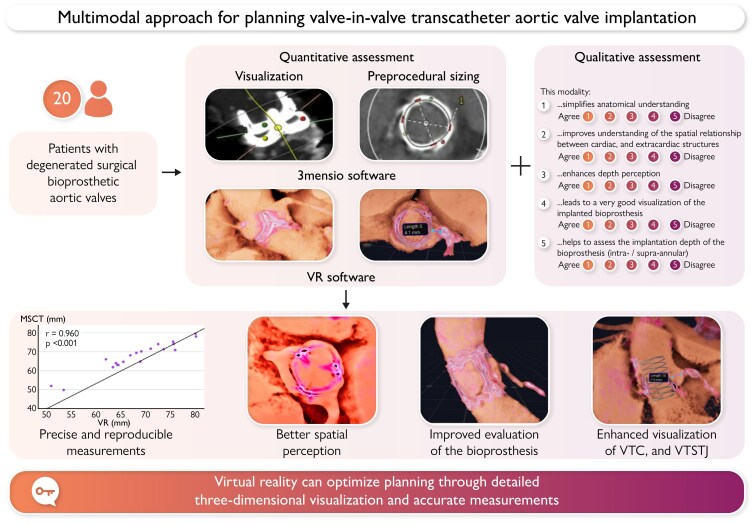
Combining standard methods and modern technologies such as virtual reality can potentially optimize the pre-procedural planning of valve-in-valve transcatheter aortic valve implantation procedures. VR, virtual reality; VTC, virtual valve to coronary ostium; VTSTJ, virtual valve to sinotubular junction.

## Introduction

Transcatheter aortic valve implantation (TAVI) has proven to be an effective treatment option not only for patients with degenerated native aortic valves but also for patients with failed surgical aortic bioprosthesis.^1^ Valve-in-valve (ViV)-TAVI is becoming more and more a relevant treatment alternative to redo surgical aortic valve replacement. It was observed that ViV-TAVI resulted in lower in-hospital mortality, and the risks of stroke, post-procedure pacemaker implantation, major adverse cardiac events, and mortality during 30-day and 6-month readmissions were comparable with those associated with repeat surgical aortic valve replacement.^2^ Although less invasive than surgical reoperation, ViV-TAVI treatment may be challenging.^3^ Thus, precise patient selection and individualized pre-procedural planning are essential for procedural success and optimal long-term outcomes.^4^ High-quality imaging plays a key role in this process. Accurate visualization and measurements of the patient’s anatomy and the condition of the existing bioprosthetic valve are crucial for determining the appropriate size and type of new valve to be implanted.^5^ Due to its high-resolution images, multislice computed tomography (MSCT) is considered the gold standard for pre-procedural planning. Multislice computed tomography enables the determination of the dimensions of the bioprosthetic valve and allows the assessment of calcium expansion and distribution within and around the bioprosthetic valve. In addition, it also helps to plan the access route and evaluate the relationship between the coronary ostia and the bioprosthetic valve.^6^ However, even though the MSCT images and reconstructions are three-dimensional (3D), they are still presented on two-dimensional screens.^7^ This may impair depth perception and the understanding of complex 3D anatomical relationships. Virtual reality (VR) is an innovative tool that could help overcome these challenges. Using VR to create a 3D visualization of the patient’s specific anatomy provides a new perspective for perceiving and interpreting the data.^8^ As a result, there are a growing number of studies investigating the use of VR in planning interventions across various medical fields.^9^ We have already demonstrated the feasibility of using VR for pre-procedural planning of TAVI procedures.^10^ To advance the field, the next step was to evaluate the VR application during the planning of ViV-TAVI as a more complex scenario. This study investigated the feasibility, accuracy, and reproducibility of visualizing and measuring the surgical aortic bioprostheses and their surrounding structures in VR. Furthermore, we also examined the potential benefits this modality can offer for pre-procedural planning.

## Methods

### Study design and population

This study retrospectively included 20 non-consecutive patients with symptomatic severe stenosis of the stented bioprosthetic aortic valve who had undergone ViV-TAVI at Heart Center Duesseldorf. Patients with a stentless bioprosthetic aortic valve were excluded. Valve-in-valve-transcatheter aortic valve implantation was indicated based on the severity of stenosis determined by transthoracic echocardiography, transoesophageal echocardiography, and the local heart team’s recommendation. Pre-procedural planning was carried out with the 3mensio software following the standard operating procedures based on MSCT data. Procedural data were collected from reports and imaging data. The length of the prosthesis below the native annulus was measured for assessment of implantation depth. All procedures were performed in accordance with the Declaration of Helsinki, and the local ethics committee of the University Hospital Duesseldorf approved the study protocol (IRB approval no. 2022-1946). All patients signed the informed consent to participate in this study.

### Multislice computed tomography and 3mensio software measurements

All patients underwent pre-procedural high-resolution MSCT. A single-source computed tomography scanner (SOMATOM Definition Edge, Siemens Healthineers, Erlangen, Germany) with an electrocardiogram triggering was used to acquire the images (128 × 0.6 mm collimation, 142 ms temporal resolution, 300 ms rotation time, and automated tube current adaption). The mid-systolic phase was used to reconstruct axial images with a slice thickness of 0.75 mm, and all patients received 80 mL of contrast medium for the performance of MSCT angiography. The image data sets were saved as Digital Imaging and Communications in Medicine (DICOM) files. All data were analysed, and measurements were performed by a highly experienced physician in this field in accordance with best practice recommendations using dedicated software (3mensio Structural Heart™, Pie Medical Imaging BV, Maastricht, The Netherlands).^11^

### Visualization of the data in virtual reality

Virtual reality offers the user a 3D immersion experience by using a headset covering the entire field of view and controllers that allow interaction with the virtual world. In our study, we used the Meta Quest 2 (Meta, Irvine, CA, USA) headset with a resolution of 20 pixels per degree, a refresh rate of 120 Hz, and a fast-switch LCD Display with 1832 × 1920 pixels per eye. Furthermore, by using two controllers with three rotational movements and three translational movements, and the ability to zoom, grip, cut, and rotate the heart and surrounding structures, it was possible to view the anatomy and the bioprosthesis in detail and any desired plane. The VR models were created based on the MSCT images using dedicated software (VMersive and Vea Simulations, Warsaw, Poland).

### Valve-in-valve-transcatheter aortic valve implantation sizing protocol for virtual reality

**Figure 1:**
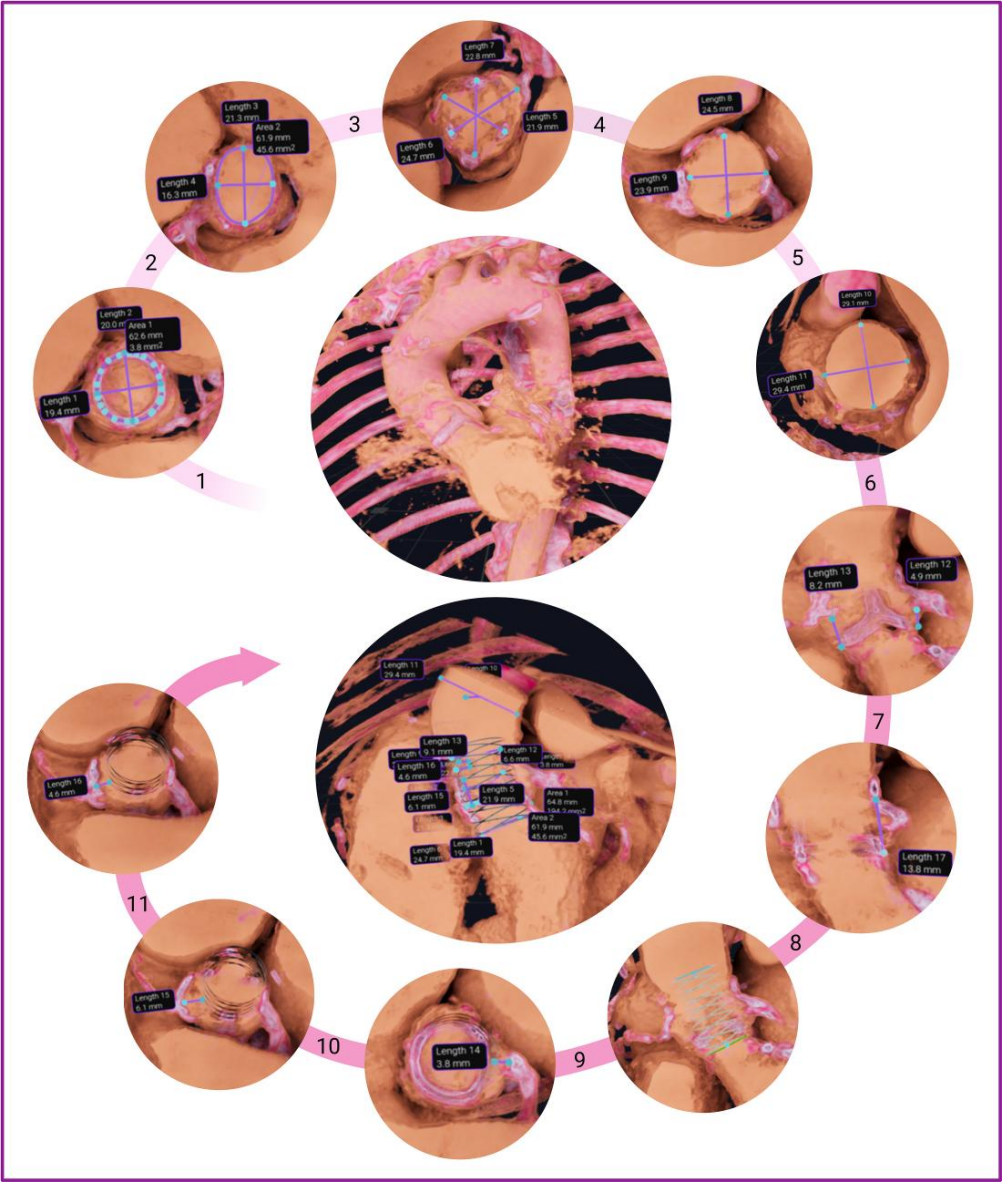
Sizing protocol for valve-in-valve-transcatheter aortic valve implantation in virtual reality. In the first step, the bioprosthesis was visualized in a cross-sectional view and the annulus plane was measured (1). Next, 5 mm distal to the bioprosthesis measurements of the left ventricular outflow tract were taken (2). Afterward, a plane was positioned through the sinus of Valsalva for cross-sectional measurements (3). The sinotubular diameter was measured in a transverse, double oblique plane aligned with the sinotubular junction (4), and the ascending aorta 4 cm above the bioprosthesis (5). Following, a vertical line connecting the lower edge of the orifice of each coronary artery and the lowest edge of the sinotubular junction to the plane of the bioprosthesis was used to measure the distance between the coronary arteries (6) and the sinotubular junction (7). In the next step, with the help of the ViV Aortic App, a virtual cylinder was inserted into the bioprosthesis (8), and the distances to the coronary ostium left (9) and right (10), as well as to the sinotubular junction (11) were measured.

After uploading the MSCT DICOM data to the VR software, an automatic 3D reconstruction was performed, and an initial rough orientation was obtained by viewing the entire model. After acquiring an initial overview, a closer look was taken at the structures by visualizing the anatomy in the various image profiles available and applying the abovementioned functions. All measurements were conducted in the same phase (best mid-systole) according to a protocol developed for this purpose, similar to MSCT measurements (*Figure 1*). Three physicians without knowing the 3mensio measurements carried out the measurements. Two had prior VR experience (over 100 measurements each), while the third person conducted the measurements following a training session. In the first step, the bioprosthesis was visualized in an oblique plane by moving the virtual heart in the sagittal, coronal, and axial planes. Then, after zooming in and cutting out the adjacent structures using the virtual tool, the bioprosthesis was presented in a cross-sectional plane to measure the annulus plane, represented by the basal ring. Next, the left ventricular outflow tract (LVOT) was measured similarly to before the TAVI procedures. A straight line was drawn from the plane’s centre towards the LVOT, and measurements were taken 5 mm distal to the bioprosthesis. Following, a plane was positioned through the sinus of Valsalva (SoV) for cross-sectional measurements. Afterward, the sinotubular diameter was measured in a transverse, double oblique plane aligned with the sinotubular junction, and 4 cm above the bioprosthesis of the ascending aorta. The distance to the coronary arteries and the sinotubular junction (STJ) was measured by drawing a vertical line connecting the lower edge of the orifice of each coronary artery and the lowest edge of the STJ to the plane of the bioprosthesis. In the last step, we used a dedicated app (ViV Aortic App, London, UK) to determine the true internal diameter (ID), which differs from the stent ID.^12^ Stent ID is based on the stent diameter without leaflets, whereas true ID is the inner diameter of the valve with leaflets. This is the most important dimension when selecting the size of the transcatheter heart valve (THV) implanted in a given bioprosthesis.^13^ The true ID data were used to create a virtual cylinder inserted into the bioprosthesis. Subsequently, the distances of the virtual cylinder to the coronary ostium right and left as well as the distance to the sinotubular junction (VTSTJ) were measured.

### Qualitative assessment

Additionally, eight interventionalists evaluated the use of VR for planning ViV-TAVI procedures. A structured questionnaire was created for this purpose and used to assess both VR and 3mensio software to determine the strengths and weaknesses of each modality. Aspects such as depth perception, visualization of the prosthesis, and adjacent structures, as well as access routes were assessed using a 5-point Likert scale, where 1 means ‘I strongly agree’ and 5 means ‘I strongly disagree’.

### Statistical analysis

Analyses were performed using IBM SPSS Statistics version 29 for Windows (IBM Corp., Armonk, NY, USA). Shapiro–Wilk tests were used to assess the normal distributions among the analysed data. Continuous variables were shown as mean ± standard deviation, or as median and interquartile range in cases of non-normal distribution. Frequency and percentage were used to summarize categorical variables. Data with normally distributed samples were compared using the Student’s two-sample *t*-test, and data with non-normally distributed samples were compared using the Wilcoxon test. For assessing agreement between the methods (3mensio and VR), the Bland–Altman plot was used. Pearson’s correlations were applied to evaluate linear and monotonic relationships between the methods. Statistical significance was assumed, if the *P*-value was <0.05.

Experienced imaging specialists performed all measurements to analyse the interobserver variability. To analyse the intraobserver reliability 1 physician repeated the measurements of 10 patients 3 months later. Inter- and intraobserver variability for VR and MSCT measurements was evaluated using the intraclass correlation coefficient (ICC). Inter- and intraobserver reliability was interpreted as excellent for ICC >0.90, good between 0.75 and 0.90, moderate between 0.50 and 0.75, and poor <0.50.^14^

## Results

### Study population

Twenty patients with severe stenosis of the stented bioprosthetic aortic valve, who successfully underwent ViV-TAVI at the Heart Center Duesseldorf, were included. All the required data were available to conduct a thorough analysis. Patients had a mean age of 75 ± 8 years, and 75% were male (15 patients). The most common comorbidities included heart failure (all stages from heart failure with preserved ejection fraction to heart failure with reduced ejection fraction) (90%), arterial hypertension (85%), and chronic kidney disease (75%). Five patients (25%) had atrial fibrillation. All baseline characteristics are displayed in *Table 1*. A majority of patients (65%) had an Edwards Perimount Magna Ease prosthesis, and on average, the failed prostheses were 9 ± 5 years old. Detailed information are summarized in Supplementary material online, *Table S1*. Furthermore, the procedure data are presented in Supplementary material online, *Table S2*.

**Table 1:** Baseline characteristics

Patient characteristics	*N* = 20
Male gender, *n* (%)	15 (75%)
Age, M (years) ± SD	75 ± 9
Height, M (m) ± SD	1.74 ± 0.1
Weight, M (kg) ± SD	82.3 ± 18.4
BMI, M (kg/m^2^) ± SD	27.5 ± 8.8
BSA (m^2^) ± SD	1.98 ± 0.2
Arterial hypertension, *n* (%)	17 (85%)
Pulmonary hypertension	4 (20%)
Atrial fibrillation, *n* (%)	5 (25%)
Diabetes mellitus, *n* (%)	10 (50%)
Heart failure	18 (90%)
s/p. MI	8 (40%)
s/p. CABG	8 (40%)
s/p. PCI, *n* (%)	8 (40%)
s/p. valvuloplasty, *n* (%)	0 (0%)
s/p. CVA, *n* (%)	3 (15%)
CKD, *n* (%)	15 (75%)
COPD, *n* (%)	6 (30%)
Current smokers, *n* (%)	4 (20%)
PVD, *n* (%)	6 (30%)
NYHA, M (m) ± SD	3 ± 1
CCS, M (m) ± SD	1 ± 1
CHA_2_DS_2_-VASc score, M ± SD	3 ± 1
HAS-BLED score, M ± SD	4 ± 1
EuroScore II, M ± SD	8.5 ± 5.6
STS score	4.4 ± 3.0

Overview of clinical patient characteristics. Values are presented as mean ± SD or expressed in *n* (%).

BMI, basal metabolic index; BSA, body surface area; CABG, coronary artery bypass grafting; CCS, Canadian Cardiovascular Society; CKD, chronic kidney disease; COPD, chronic obstructive pulmonary disease; CVA, cerebrovascular accident; NYHA, New York Heart Association; M, mean; MI, myocardial infarction; PCI, percutaneous coronary intervention; PVD, peripheral vascular disease; SD, standard deviation.

### Virtual reality and 3mensio measurements

**Figure 2:**
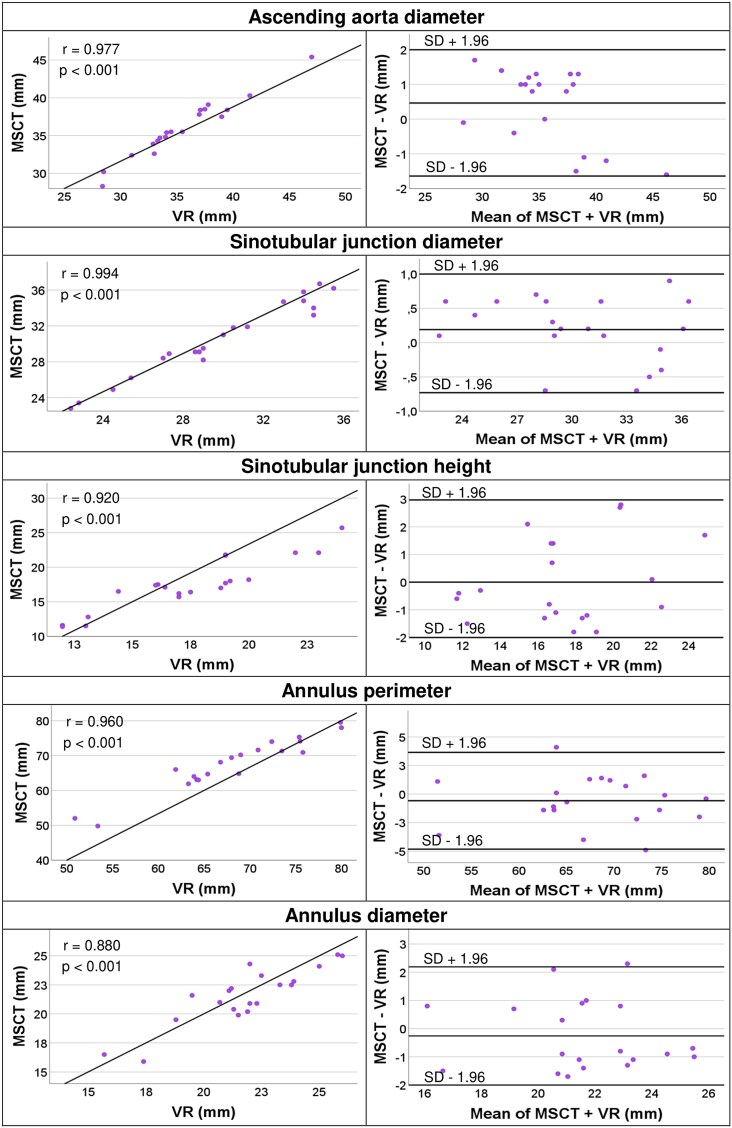
Comparison of virtual reality and 3mensio measurements. Scatterplots and Bland–Altman plots show good or excellent correlation and agreement of all collected virtual reality measurements compared to 3mensio measurements. M, mean; MSCT, multislice computed tomography; SD, standard deviation; *r*, Pearson correlation; VR, virtual reality.

Due to the sufficient quality of the MSCT images, visualization and measurement with VR software were feasible for all cases. The analysis showed no significant differences between the VR and 3mensio measurements. All collected results are presented in *Table 2*. The degree of correlations is shown using scatterplots and Bland–Altman plots (*Figure 2*). The *P*-value for all correlations was <0.001.

**Table 2:** Comparison between conventional multislice computed tomography assessment and virtual reality measurements

Variable	Conventional MSCT assessment	VR	*P*-value
AA min diameter	34.77 ± 3.68	34.21 ± 4.39	0.057
AA max diameter	36.73 ± 4.05	36.37 ± 4.58	0.242
AA mean diameter	35.77 ± 3.84	35.31 ± 4.45	0.068
STJ min diameter	29.63 ± 4.17	29.47 ± 4.31	0.311
STJ max diameter	31.40 ± 4.35	31.16 ± 4.37	0.089
STJ mean diameter	30.53 ± 4.20	30.34 ± 4.31	0.086
STJ height	17.42 ± 3.87	17.42 ± 3.44	0.988
Annulus perimeter	67.59 ± 7.64	68.17 ± 7.72	0.241
Annulus min diameter	20.82 ± 2.35	20.78 ± 2.82	0.910
Annulus max diameter	22.19 ± 2.63	22.75 ± 2.53	0.055
Annulus mean diameter	21.53 ± 2.45	21.79 ± 2.60	0.372
LVOT perimeter	76.41 ± 9.61	75.87 ± 8.67	0.242
LVOT min diameter	22.63 ± 2.87	22.36 ± 2.76	0.209
LVOT max diameter	26.08 ± 3.54	25.59 ± 2.97	0.209
LVOT mean diameter	24.38 ± 2.99	23.99 ± 2.68	0.105
SoV L diameter	31.70 ± 4.66	31.18 ± 4.50	0.061
SoV R diameter	30.74 ± 4.64	30.26 ± 4.16	0.085
SoV NC diameter	31.40 ± 5.05	31.47 ± 5.07	0.700
RCA height	10.31 ± 3.85	10.51 ± 3.70	0.306
LCA height	6.02 ± 3.89	6.20 ± 3.88	0.232
VTC left	5.54 ± 1.97	5.79 ± 1.85	0.072
VTC right	5.61 ± 1.15	5.71 ± 1.30	0.508
VTSTJ	5.74 ± 0.89	5.61 ± 1.20	0.330

Values are presented as mean ± SD in millimetres.

AA, ascending aorta; L, left; LCA, left coronary artery; LVOT, left ventricular outflow tract; max, maximum; min, minimum; MSCT, multislice computed tomography; NC, non-coronary; R, right; RCA, right coronary artery; SoV, sinus of Valsalva; STJ, sinotubular junction; VR, virtual reality; VTC, virtual valve to coronary ostium; VTSTJ, virtual valve to sinotubular junction.

### Inter- and intraobserver reliability

Good-to-excellent interobserver agreement was documented for all values. The ICC values for VR measurements were between 0.897 and 0.986 (*Table 3*), and for MSCT measurements between 0.937 and 0.998 (Supplementary material online, *Table S3*). Similar results were achieved for intraobserver reliability. The values are shown in *Table 4* and Supplementary material online, *Table S4*.

**Table 3:** Interobserver reliability of virtual reality measurements

Variable	Interobserver reliability (*n* = 20)			
	Investigator 1	Investigator 2	Investigator 3	ICC (95% CI)
AA diameter min	34.21 ± 4.39	34.60 ± 4.08	34.84 ± 4.22	0.975 (0.948–0.989)
AA diameter max	36.37 ± 4.58	36.58 ± 4.24	36.99 ± 4.57	0.980 (0.958–0.991)
AA diameter mean	35.31 ± 4.45	35.61 ± 4.13	35.94 ± 4.33	0.982 (0.961–0.992)
STJ diameter min	29.47 ± 4.31	29.66 ± 4.50	29.38 ± 4.37	0.977 (0.951–0.990)
STJ diameter max	31.16 ± 4.37	31.20 ± 4.55	31.15 ± 4.51	0.977 (0.952–0.990)
STJ diameter mean	30.34 ± 4.31	30.46 ± 4.49	30.28 ± 4.42	0.978 (0.954–0.991)
STJ height	17.42 ± 3.44	17.53 ± 3.23	18.19 ± 3.66	0.976 (0.945–0.990)
Annulus perimeter	68.17 ± 7.72	67.83 ± 8.22	67.81 ± 7.69	0.986 (0.970–0.994)
Annulus diameter min	20.78 ± 2.82	20.75 ± 3.09	20.73 ± 2.87	0.951 (0.897–0.979)
Annulus diameter max	22.75 ± 2.53	22.64 ± 3.05	22.54 ± 2.70	0.947 (0.888–0.977)
Annulus diameter mean	21.79 ± 2.60	21.72 ± 3.03	21.65 ± 2.76	0.950 (0.894–0.979)
LVOT perimeter	75.87 ± 8.67	75.69 ± 9.09	76.59 ± 8.24	0.985 (0.969–0.994)
LVOT diameter min	22.36 ± 2.76	21.97 ± 2.95	22.45 ± 2.34	0.950 (0.896–0.979)
LVOT diameter max	25.59 ± 2.97	25.36 ± 3.12	26.00 ± 2.87	0.922 (0.838–0.967)
LVOT diameter mean	23.99 ± 2.68	23.68 ± 2.89	24.26 ± 2.44	0.931 (0.857–0.970)
SoV L diameter	31.18 ± 4.50	31.35 ± 4.12	31.41 ± 4.31	0.980 (0.958–0.991)
SoV R diameter	30.26 ± 4.16	30.52 ± 3.91	31.12 ± 3.94	0.981 (0.956–0.992)
SoV NC diameter	31.47 ± 5.07	31.58 ± 4.90	31.06 ± 4.81	0.985 (0.969–0.994)
RCA height	10.51 ± 3.70	10.68 ± 3.61	11.09 ± 3.84	0.983 (0.964–0.993)
LCA height	6.20 ± 3.88	6.58 ± 4.20	7.05 ± 3.82	0.986 (0.965–0.995)
VTC left	5.79 ± 1.85	5.90 ± 1.83	5.58 ± 1.71	0.961 (0.918–0.983)
VTC right	5.71 ± 1.30	5.92 ± 1.19	5.81 ± 1.30	0.913 (0.817–0.963)
VTSTJ	5.61 ± 1.20	6.07 ± 1.21	5.65 ± 1.03	0.865 (0.716–0.942)

Values are presented as mean ± SD in millimetres. ICC for measurements for interobserver agreement are displayed in the table.

AA, ascending aorta; L, left; LCA, left coronary artery; LVOT, left ventricular outflow tract; max, maximum; min, minimum; MSCT, multislice computed tomography; NC, non-coronary; R, right; RCA, right coronary artery; SoV, sinus of Valsalva; STJ, sinotubular junction; VR, virtual reality; VTC, virtual valve to coronary ostium; VTSTJ, virtual valve to sinotubular junction.

**Table 4:** Intraobserver reliability of virtual reality measurements

Variable	Intraobserver reliability (*n* = 10)		
	1. Measurement	2. Measurement	ICC (95% CI)
AA diameter min	35.65 ± 5.39	35.30 ± 5.46	0.995 (0.981–0.999)
AA diameter max	37.62 ± 5.30	37.10 ± 5.17	0.994 (0.959–0.999)
AA diameter mean	36.65 ± 5.34	36.24 ± 5.32	0.995 (0.976–0.999)
STJ diameter min	30.08 ± 4.52	29.77 ± 4.69	0.994 (0.979–0.998)
STJ diameter max	31.44 ± 4.39	31.34 ± 4.62	0.992 (0.970–0.998)
STJ diameter mean	30.80 ± 4.43	30.57 ± 4.63	0.993 (0.975–0.998)
STJ height	16.95 ± 3.30	16.90 ± 3.19	0.992 (0.969–0.998)
Annulus perimeter	68.54 ± 6.75	67.93 ± 7.06	0.988 (0.953–0.997)
Annulus diameter min	20.54 ± 2.45	20.09 ± 2.30	0.949 (0.805–0.987)
Annulus diameter max	22.54 ± 2.44	22.33 ± 2.70	0.980 (0.925–0.995)
Annulus diameter mean	21.57 ± 2.38	21.23 ± 2.44	0.968 (0.878–0.992)
LVOT perimeter	74.85 ± 8.18	74.43 ± 8.34	0.995 (0.981–0.999)
LVOT diameter min	22.13 ± 2.96	21.83 ± 3.02	0.984 (0.937–0.996)
LVOT diameter max	25.00 ± 2.40	24.85 ± 2.49	0.982 (0.932–0.996)
LVOT diameter mean	23.57 ± 2.57	23.35 ± 2.64	0.982 (0.932–0.995)
SoV L diameter	31.30 ± 4.39	31.17 ± 4.59	0.995 (0.982–0.999)
SoV R diameter	31.11 ± 3.82	30.93 ± 3.84	0.996 (0.983–0.999)
SoV NC diameter	31.45 ± 4.65	31.22 ± 4.70	0.996 (0.983–0.999)
RCA height	11.42 ± 3.97	11.51 ± 3.71	0.992 (0.970–0.998)
LCA height	7.78 ± 4.13	8.03 ± 3.92	0.992 (0.969–0.998)
VTC left	5.85 ± 1.63	6.19 ± 1.26	0.912 (0.668–0.978)
VTC right	5.56 ± 1.38	5.88 ± 1.21	0.958 (0.771–0.990)
VTSTJ	5.60 ± 1.18	5.85 ± 1.13	0.975 (0.800–0.995)

Values are presented as mean ± SD in millimetres. ICC for measurements for intraobserver agreement are displayed in the table.

AA, ascending aorta; L, left; LCA, left coronary artery; LVOT, left ventricular outflow tract; max, maximum; min, minimum; MSCT, multislice computed tomography; NC, non-coronary; R, right; RCA, right coronary artery; SoV, sinus of Valsalva; STJ, sinotubular junction; VR, virtual reality; VTC, virtual valve to coronary ostium; VTSTJ, virtual valve to sinotubular junction.

### Qualitative comparison of both methods

**Figure 3:**
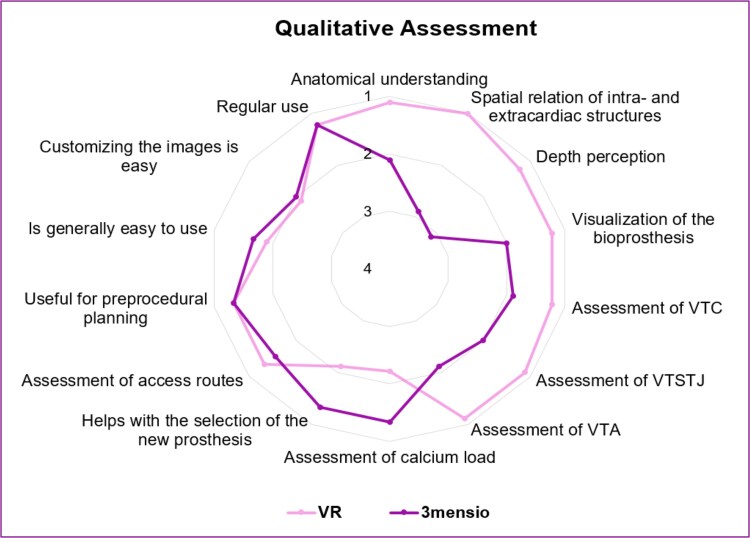
Qualitative assessment of virtual reality and 3mensio software. The radar chart displays results from the structured questionnaire with mean values from 1 (strongly agree) to 5 (strongly disagree). Due to the three-dimensional visualization, virtual reality offers advantages in depth perception and the assessment of distances between the prosthesis and coronaries, sinotubular junction, and aorta. Multislice computed tomography remains the method of choice for assessing the calcification load and selecting a new prosthesis. MSCT, multislice computed tomography; VR, virtual reality; VTA, virtual valve to aorta, VTC, virtual valve to coronary ostium; VTSTJ, virtual valve to sinotubular junction.

The analyses confirmed that VR gives the users a better understanding of the spatial relationships between the cardiac and extracardiac structures (1.0 ± 0 vs. 2.9 ± 0.8, *P* = 0.007), and the 3D visualization can optimize depth perception (1.2 ± 0.4 vs. 3.1 ± 1.0, *P* = 0.007). Furthermore, VR enabled excellent visualization of the bioprosthesis (1.2 ± 0.4 vs. 2.0 ± 0.5, *P* = 0.020) and was found to be a better method to visualize the distances between the virtual prosthesis to the coronaries (1.1 ± 0.3 vs. 2.0 ± 0.7, *P* = 0.023), STJ (1.2 ± 0.4 vs. 1.9 ± 0.6, *P* = 0.034), and (1.1 ± 0.3 vs. 2.1 ± 0.8, *P* = 0.014) (*Graphical abstract*). Moreover, although the differences were not significant, VR also proved to be a good method for evaluating access routes (1.3 ± 0.5 vs. 1.6 ± 0.7, *P* = 0.414), and both software programs were rated as user-friendly (1.9 ± 0.9 vs. 1.7 ± 0.5, *P* = 0.317). However, there were no significant differences in the prediction of the risk of coronary occlusion and the use of coronary protection strategies [e.g. bioprosthetic or native aortic scallop intentional laceration to prevent iatrogenic coronary artery obstruction (BASILICA) or chimney–stenting technique] (2.2 ± 0.8 vs. 2.0 ± 0.7, *P* = 0.157), and 3mensio was the preferred method for selecting the new prosthesis (2.1 ± 0.6 vs. 1.3 ± 0.5, *P* = 0.038). In general, the participants found both methods helpful in preparing for interventions (1.3 ± 0.5 vs. 1.3 ± 0.5, *P* = 1.000), and they could imagine using them regularly (1.2 ± 0.7 vs. 1.2 ± 0.4, *P* = 1.000; *Figure 3*).

## Discussion

The present study evaluated for the first time the use of VR for pre-procedural planning of ViV-TAVI procedures.

Our main findings were as follows:

- The application of VR for detailed visualization of stented bioprosthetic aortic valves is feasible.
- Pre-procedural sizing can be performed using VR, and the values do not differ significantly from 3mensio measurements.
- The results demonstrated high levels of reliability for both intra- and interobserver assessments.
- In addition to improving the 3D understanding of patient anatomy, VR helps in the assessment of access routes and ViV-specific aspects like the distance of the virtual heart valve to the coronaries and the sinotubular junction.

Rapid advancements in structural imaging and simulation technologies have opened up new possibilities for planning and performing interventions in the field of structural heart disease.^15,16^ By visualizing anatomy in immersive ways and delivering detailed patient-specific insights, VR, augmented reality, 3D printing, and holograms are increasingly being used for this purpose.^17^ Its use for planning procedures, such as closure of the left atrial appendage,^18^ TAVI,^19^ transcatheter closure of paravalvular leaks,^20^ MitraClip intervention,^21^ or closure of atrial septal defects,^22^ has already been evaluated. The models were generated using MSCT or magnetic resonance imaging (MRI) data, enabling a detailed examination of the heart's structure, including the valves, chambers, and blood vessels. This not only led to a better understanding of the anatomy and pathology but also enabled the recognition of important aspects not directly visible on the MSCT/MRI images. However, many of these studies focus on qualitative aspects, and there is a lack of quantitative data. The current data on the use of VR for planning ViV-TAVI procedures are still very limited. One study assessed the benefits of VR in personalized scenarios for TAVI planning.^23^ In 2 out of 11 cases, a ViV-TAVI procedure was performed and in both cases, VR models modified the implant strategy. In both patients, the MSCT and 3D measurements showed a small distance to the coronary arteries. The VR visualization could help interventionalists to precisely assess the distance and thus facilitate the decision of whether coronary protection is necessary. However, the exact measurements were not documented. In general, the pre-procedural use of VR has improved self-assurance in all cases. The planning of ViV-TAVI procedures can be challenging, because surgical bioprostheses are very different and vary in their tissue characteristics, frame designs, and implantation methods. To determine the feasibility of ViV-TAVI and to plan the procedure, knowledge of the surgical bioprosthesis is essential. Therefore, a detailed presentation with the recording of all characteristics and precise measurements is crucial to prevent the feared complications, such as malposition, coronary impairment, or prosthesis–patient mismatch.^24^ Our study has shown that it is feasible to visualize the bioprostheses in VR based on MSCT data. Furthermore, for the first time, we have demonstrated that it is possible to perform pre-procedural sizing using VR and that the measurements correspond well with the measurements taken with the standard software. Another dreaded complication is coronary artery obstruction.^25^ Valve-in-valve-transcatheter aortic valve implantation is known to have a higher risk of coronary obstruction than native TAVI and imaging plays a major role in understanding the 3D interaction between the degenerated bioprosthesis and coronary arteries ostia to minimize this risk and preserve future coronary access.^26^ The parameters that help with the risk assessment include the distances between the THV and the coronary arteries as well as the STJ.^27^ Furthermore, they are also used in the Valve-in-Valve International Data classification, which helps interventionalists to assess whether protection using chimney stenting or BASILICA would be necessary.^28^ With the help of the VR software, it was possible to insert a virtual cylinder and the qualitative analysis showed that the visualization of the distances was better in VR than in 3mensio. This can be attributed to the 6 degrees of freedom that allow us to freely move the virtual heart in 3D and view the cardiac and extracardiac structures from different directions and angles, which can improve the evaluation of spatial relationships. Here again, the VR measurements did not show significant differences compared to the 3mensio measurements. However, only stented bioprostheses were evaluated in this study. It would be interesting to evaluate whether other procedures such as TAVI-in-TAVI interventions could benefit from the use of VR. The assessment of the degree of expansion of the index THV, the implantation depth, the risk plane, or the neoskirt can sometimes be challenging with MSCT.^29^ The use of VR could also provide additional useful information in this area. In addition, we could also show that VR can be a good tool for evaluating access routes. Patients who receive a ViV-TAVI procedure often have peripheral vascular disease so it is important to look closely at the access routes to prevent complications such as bleeding, vascular occlusion, or aneurysm.^30,31^ By providing a 3D representation, VR enables a more precise understanding of the course of the vessels and can help to identify potential difficulties at an early stage. However, due to the limited data available and existing uncertainty about the extent to which the use of technology can improve the complication rate, treatment success, and quality of life of patients, the users favoured MSCT for the selection of the prosthesis.

Our data were collected retrospectively and the monocentric nature of the study limits the generalization of the data due to selection bias. Randomized, prospective, multicentre studies are needed to better assess the impact on improving clinical outcomes. Due to the different strengths and weaknesses of each modality, participants reported that both methods can be useful for pre-procedural preparation. At a time when personalized medicine is playing an increasingly important role, a combination of various imaging techniques is essential to encompass all patient-specific aspects. Innovative technologies such as VR or augmented reality are developing very quickly and are becoming increasingly user-friendly. It is expected that soon it will not only be possible to perform pre-procedural planning in VR but also to simulate complete procedures, such as TAVI. However, even though more and more studies on the application of new innovative technologies in medicine are showing promising results, implementation in everyday clinical practice is still challenging. The cost of implementing VR hardware, software, and the necessary infrastructure can be prohibitive for many healthcare institutions. Moreover, many physicians have never used these technologies before and would need to be properly trained to use these technologies effectively. It can be time-consuming to introduce new technologies in clinical routine, which can lead to skepticism and reluctance among physicians. However, if these challenges can be overcome and more data becomes available, these technologies have great potential to significantly improve everyday clinical practice.

In summary, there is no objective difference between measurements performed by imaging specialists using VR and standard MSCT assessment, as both methods have similar measurement accuracy. However, the key difference lies in the subjective benefit of a better understanding of the anatomy by the interventionalists. Virtual reality seems to offer a more intuitive and immersive method of visualizing and interacting with anatomical structures, thereby improving clinicians’ comprehension and confidence in clinical decision-making.

The current studies primarily focus on the accuracy and reproducibility of measurements, but these metrics do not correlate with patient outcomes directly. Therefore, future research should focus on more clinically relevant endpoints, such as complication rates, procedural success, patient recovery times, and long-term health outcomes, to better assess whether the use of VR truly improves patient care.

## Conclusions

Imaging is an integral part of the planning of ViV-TAVI procedures, from patient selection, choosing the right valve size and type, assessing vascular access to identifying potential risks and finally performing the procedure. Our study has shown that the use of VR for ViV-TAVI planning is feasible and VR allows accurate pre-procedural sizing. Furthermore, VR offers additional benefits, including a detailed representation of the bioprosthesis and improved analysis of anatomical relationships. This can provide new insights for planning the procedure and can be used in a complementary way, particularly when assessing degenerated bioprostheses, which is often very complex. This multimodal approach can enable a comprehensive assessment, which may contribute to the success and safety of the procedure and potentially enhance the patient's treatment.

## Supplementary Material

ztaf024_Supplementary_Data

## Data Availability

The anonymized data can be requested from the authors if required.
