# Supplementary material for: Virtual reality for pre-procedural planning of valve-in-valve transcatheter aortic valve implantation
Source: Eur Heart J Digit Health. 2025 Mar 25;6(3):372–81. doi: 10.1093/ehjdh/ztaf024 (PMC12088716; doi:10.1093/ehjdh/ztaf024)
Supplement: ztaf024_Supplementary_Data [file ztaf024_supplementary_data.docx]

**Table S1:** Overview of SAV bioprosthesis and the implantation years

| **Patient** | **SAV bioprosthesis** | **Implantation year** |
| --- | --- | --- |
| 1. | Edwards Perimount Magna Ease | 2016 |
| 2. | Medtronic ATS Enable | 2011 |
| 3. | Edwards Perimount Magna Ease | 2013 |
| 4. | Abbott Trifecta | 2013 |
| 5. | Edwards Inspiris | 2020 |
| 6. | Edwards Perimount Magna Ease | 2013 |
| 7. | Edwards Perimount Magna Ease | 2016 |
| 8. | Edwards Perimount Magna Ease | 2009 |
| 9. | Edwards Perimount Magna Ease | 2011 |
| 10. | Abbott Epic Bicor | 1998 |
| 11. | Medtronic Hancock | 2014 |
| 12. | Edwards Perimount Magna Ease | 2009 |
| 13. | Edwards Perimount Magna Ease | 2011 |
| 14. | Edwards Perimount Magna Ease | 2014 |
| 15. | Sorin Mitroflow | 2008 |
| 16. | Edwards Perimount Magna Ease | 2015 |
| 17. | Edwards Perimount Magna Ease | 2016 |
| 18. | Sorin Mitroflow | 2010 |
| 19. | Edwards Perimount Magna Ease | 2014 |
| 20. | Edwards Perimount Magna Ease | 2004 |

**Table S2:** Overview of procedural data

| **Procedural data** | **N = 20** |
| --- | --- |
| Procedure time (min) | 93.6 ± 31.6 |
| Fluoroscopy time (min) | 23.1 ± 11.9 |
| Contrast volume (ml) | 53.9 ± 20.7 |
| Dose area product (cGy*cm²) | 6933.1 ± 342.6 |
| Implantation depth at NCC site (mm) | - 3.1 ± 2.0 |
| Implantation depth at LCC site (mm) | - 3.8 ± 1.9 |

Values are presented as mean ± SD.

LCC: left coronary cusp; NCC: non-coronary cusp

**Table S3:** Interobserver reliability of MSCT measurements

|  | **Interobserver reliability (n =20)** | | |
| --- | --- | --- | --- |
| **Variable** | **Investigator 1** | **Investigator 2** | **ICC (95% CI)** |
| AA diameter min | 34.77 ± 3.68 | 34.61 ± 3.70 | 0.994 (0.986 – 0.998) |
| AA diameter max | 36.73 ± 4.05 | 37.18 ± 4.33 | 0.937 (0.841 – 0.975) |
| AA diameter mean | 35.77 ± 3.84 | 35.91 ± 3.84 | 0.982 (0.954 – 0.993) |
| STJ diameter min | 29.63 ± 4.17 | 29.42 ± 4.09 | 0.994 (0.986 – 0.998) |
| STJ diameter max | 31.40 ± 4.35 | 31.14 ± 4.36 | 0.995 (0.988 – 0.998) |
| STJ diameter mean | 30.53 ± 4.20 | 30.33 ± 4.20 | 0.998 (0.994 – 0.999) |
| STJ height | 17.42 ± 3.87 | 17.66 ± 3.76 | 0.994 (0.985 – 0.998) |
| Annulus perimeter | 67.59 ± 7.64 | 67.07 ± 7.29 | 0.998 (0.994 – 0.999) |
| Annulus diameter min | 20.82 ± 2.35 | 20.67 ± 2.13 | 0.967 (0.920 – 0.987) |
| Annulus diameter max | 22.19 ± 2.63 | 22.09 ± 2.42 | 0.984 (0.960 – 0.994) |
| Annulus diameter mean | 21.53 ± 2.45 | 21.42 ± 2.25 | 0.980 (0.950 – 0.992) |
| LVOT perimeter | 76.41 ± 9.61 | 76.72 ± 9.32 | 0.996 (0.990 – 0.998) |
| LVOT diameter min | 22.63 ± 2.87 | 23.12 ± 2.85 | 0.985 (0.962 – 0.994) |
| LVOT diameter max | 26.08 ± 3.54 | 26.42 ± 3.35 | 0.980 (0.950 – 0.992) |
| LVOT diameter mean | 24.38 ± 2.99 | 24.80 ± 2.89 | 0.976 (0.940 – 0.990) |
| SoV L diameter | 31.70 ± 4.66 | 32.07 ± 4.55 | 0.994 (0.984 – 0.998) |
| SoV R diameter | 30.74 ± 4.64 | 31.02 ± 4.56 | 0.994 (0.984 – 0.997) |
| SoV NC diameter | 31.40 ± 5.05 | 31.57 ± 4.92 | 0.993 (0.983 – 0.997) |
| RCA height | 10.31 ± 3.85 | 10.79 ± 3.70 | 0.993 (0.981 – 0.997) |
| LCA height | 6.02 ± 3.89 | 6.56 ± 3.78 | 0.992 (0.980 – 0.997) |
| VTC left | 5.54 ± 1.97 | 5.80 ± 1.90 | 0.983 (0.959 – 0.993) |
| VTC right | 5.61 ± 1.15 | 5.83 ± 1.24 | 0.968 (0.920 – 0.987) |
| VTSTJ | 5.74 ± 0.89 | 5.95 ± 1.11 | 0.938 (0.843 – 0.975) |

Values are presented as mean ± SD in millimeters. ICC for measurements for intraobserver agreement are displayed in the table.

AA: ascending aorta; L: left; LCA: left coronary artery; LVOT: left ventricular outflow tract; max: maximum; min: minimum; MSCT: multislice computed tomography; NC: non-coronary; R: right; RCA: right coronary artery; SoV: sinus of Valsalva; STJ: sinotubular junction; VR: virtual reality; VTC: virtual valve to coronary ostium; VTSTJ: virtual valve to sinotubular junction

**Table S4:** Intraobserver reliability of MSCT measurements

|  | **Intraobserver reliability (n = 10)** | | |
| --- | --- | --- | --- |
| **Variable** | **1. Measurement** | **2. Measurement** | **ICC (95% CI)** |
| AA diameter min | 35.32 ± 4.22 | 35.09 ± 3.77 | 0.991 (0.966 – 0.998) |
| AA diameter max | 37.64 ± 4.77 | 37.28 ± 4.58 | 0.997 (0.988 – 0.999) |
| AA diameter mean | 36.50 ± 4.47 | 36.19 ± 4.14 | 0.993 (0.973 – 0.998) |
| STJ diameter min | 30.23 ± 4.12 | 30.09 ± 4.17 | 0.995 (0.979 – 0.999) |
| STJ diameter max | 31.77 ± 4.31 | 31.31 ± 3.95 | 0.984 (0.934 – 0.996) |
| STJ diameter mean | 31.02 ± 4.18 | 30.72 ± 4.03 | 0.992 (0.969 – 0.998) |
| STJ height | 17.19 ± 3.52 | 17.52 ± 3.78 | 0.979 (0.915 – 0.995) |
| Annulus perimeter | 68.09 ± 4.72 | 68.83 ± 4.87 | 0.989 (0.957 – 0.997) |
| Annulus diameter min | 20.85 ± 1.42 | 21.11 ± 1.51 | 0.967 (0.866 – 0.992) |
| Annulus diameter max | 22.30 ± 1.74 | 22.66 ± 1.67 | 0.963 (0.853 – 0.991) |
| Annulus diameter mean | 21.60 ± 1.53 | 21.90 ± 1.57 | 0.966 (0.861 – 0.991) |
| LVOT perimeter | 76.67 ± 8.80 | 76.45 ± 8.94 | 0.996 (0.982 – 0.999) |
| LVOT diameter min | 22.74 ± 2.72 | 22.52 ± 2.86 | 0.983 (0.933 – 0.996) |
| LVOT diameter max | 25.90 ± 3.24 | 25.70 ± 3.19 | 0.985 (0.941 – 0.996) |
| LVOT diameter mean | 24.34 ± 2.80 | 24.11 ± 2.84 | 0.983 (0.932 – 0.996) |
| SoV L diameter | 32.35 ± 4.23 | 32.82 ± 4.49 | 0.981 (0.923 – 0.995) |
| SoV R diameter | 31.89 ± 4.91 | 31.62 ± 4.37 | 0.990 (0.958 – 0.997) |
| SoV NC diameter | 32.47 ± 5.18 | 31.92 ± 4.55 | 0.983 (0.932 – 0.996) |
| RCA height | 11.79 ± 3.67 | 11.55 ± 3.42 | 0.993 (0.970 – 0.998) |
| LCA height | 6.67 ± 5.02 | 6.48 ± 4.93 | 0.991 (0.966 – 0.998) |
| VTC left | 5.60 ± 1.49 | 5.43 ± 1.37 | 0.944 (0.630 – 0.972) |
| VTC right | 5.47 ± 1.26 | 5.66 ± 1.09 | 0.942 (0.766 – 0.986) |
| VTSTJ | 5.97 ± 0.86 | 5.82 ± 0.73 | 0.932 (0.728 – 0.983) |

Values are presented as mean ± SD in millimeters. ICC for measurements for intraobserver agreement are displayed in the table.

AA: ascending aorta; L: left; LCA: left coronary artery; LVOT: left ventricular outflow tract; max: maximum; min: minimum; MSCT: multislice computed tomography; NC: non-coronary; R: right; RCA: right coronary artery; SoV: sinus of Valsalva; STJ: sinotubular junction; VR: virtual reality; VTC: virtual valve to coronary ostium; VTSTJ: virtual valve to sinotubular junction
